# Meaning of Life Therapy: A Pilot Study of a Novel Psycho-Existential Intervention for Palliative Care in Cancer

**DOI:** 10.1177/00302228231209654

**Published:** 2023-10-26

**Authors:** Ana Rita Cardoso, Sónia Remondes-Costa, Elisa Veiga, Vera Almeida, José Rocha, Ricardo João Teixeira, Gerly Macedo, Manuela Leite

**Affiliations:** 1 Casa de Saúde do Telhal, 467980 Instituto São João de Deus, Lisboa, Portugal; 2 Department of Education and Psychology, School of Human and Social Sciences, 56066 University of Trás-os-Montes e Alto Douro, Vila Real, Portugal; 3 Faculty of Education and Psychology, Universidade Católica Portuguesa, Research Centre for Human Development, Porto, Portugal; 4 Department of Social and Behavioural Sciences, 166433 University Institute of Health Sciences(IUCS), CESPU, Gandra, Portugal; 5 UNIPRO, Oral Pathology and Rehabilitation Research Unit, 166433 University Institute of Health Sciences(IUCS), CESPU, Gandra, Portugal; 6 CINEICC- Faculty of Psychology and Education Sciences, 37829 University of Coimbra, Coimbra, Portugal; 7 REACH - Mental Health Clinic, Porto, Portugal; 8 Clinical and Health Psychology Unit, Psychiatry and Mental Health Service, 70893 Hospital da Senhora de Oliveira de Guimarães, Guimarães, Portugal; 9 iHealth4Well-being - Innovation in Health and Well-Being, Research Unit, Instituto Politécnico de Saúde do Norte, CESPU, Penafiel, Portugal

**Keywords:** meaning of life therapy, psycho-existential interventions, palliative care, dignity, cancer

## Abstract

Intervention in Palliative Care aims to provide physical, psychosocial, and spiritual relief for patients and family members. Brief interventions with a psycho-existential approach have shown positive responses; however, cultural adaptations are needed. This pilot study aimed to develop the *Meaning of Life Therapy* (MLT), a novel psycho-existential intervention, rooted in the Dignity Therapy, Life Review, and Meaning-Centered Psychotherapy. MLT was culturally adapted to the Portuguese context to include questions about forgiveness, apology, reconciliation, farewell, and a legacy document, i.e., the *Life Letter*. Nine PC cancer patients answered a 14-question MLT protocol, intended to help patients find purpose and meaning in life. Eight themes emerged: Family, Preservation of Identity, Life Retrospective, Clinical Situation, Achievements, Socio-Professional Valorization, Forgiveness/Apology/Reconciliation, and Saying Goodbye. MLT has proved its ability to respond to the psycho-existential needs of PC patients. Further studies should be conducted to gain extensive knowledge of the effectiveness of culturally responsive interventions.

## Introduction

Palliative Care (PC) is as integrated care model in health services, which seeks to promote dignity and improve the quality of life and well-being of patients and families facing chronic and progressive life-threatening illnesses (Neto, 2021). According to the World Health Organization (World Health Organization [WHO], 2002) and the Strategic Plan for the Development of Palliative Care (2021–2022), the main objective of PC is to prevent and alleviate suffering, rooted in a multidisciplinary, global and holistic approach and multiple dimensions (physical, psychological, social, spiritual, and existential), through the early identification and treatment of pain or other physical, psychosocial and spiritual needs (Associação Portuguesa de Cuidados Paliativos, 2017; European Association Palliative Care, 2018; Kon & Ablin, 2010; Neto, 2017; Ordem dos Psicólogos Portugueses [OPP], 2019).

A life-threatening illness can trigger high levels of suffering, jeopardizing one´s well-being and quality of life; its subsequent multiple losses expose a set of complex and subjective demands that pose a major challenge for health professionals (Krikorian & Limonero, 2012). PC provides not only the relief of physical symptoms, but also intends to attend to the psychosocial and spiritual aspects of the patient and family (Neto, 2021). Accordingly, Krikorian and Limonero (2012) maintain that it is paramount to design and implement interventions in a clinical context, which will approach suffering in an integral and individualized manner.

### Meeting Psychological and Spiritual Needs in Palliative Care

Emotional and spiritual suffering associated with advanced illness has a negative impact on the quality of life of PC patients (Gijsberts et al., 2019; Rainbird et al., 2009; Warth et al., 2019). Psychological and spiritual distress is often experienced by terminally ill patients, who at times develop thoughts of death and hopelessness, as well as by family members during the grieving process (Gijsberts et al., 2019; Steinhauser et al., 2000; Warth et al., 2019).

According to Harrison (2010), from an emotional standpoint, patients need to experience feelings of security, comfort and belonging to overcome the challenges of the disease. From a psychological standpoint, patients should be able to adjust to the disease and its challenges without subjective feelings of loss of control, autonomy and independence or feelings of anger and fear, which could limit adaptation to and cooperation with health care programs. On a social level, it is fundamental that the patients feel involved, accepted and recognized in their roles in the family and society. Finally, on a spiritual level, it is essential that the patients find meaning and purpose in life, strengthening their identity and values.

A recent study carried out in a palliative setting in Portugal (Antunes et al., 2020) demonstrated that psychological and spiritual care is urgent among PC patients, stressing the importance of holistic healthcare interventions.

### Psychosocial and Existential Interventions

Psychosocial and existential interventions are characterized by focusing on the psychological, social and existential needs of terminally ill patients and their families (Warth et al., 2019), aiming at preserving the patients’ dignity and helping them find meaning in life and in suffering (Caldeira et al., 2017a).

Recent systematic reviews and meta-analyses (Bauereiß et al., 2018; Huang et al., 2020; Martinez et al., 2017; Oh & Kim, 2014; Warth et al., 2019) have highlighted the association of psychosocial and existential suffering with chronic and progressive illnesses as well as the positive impact of psycho-existential interventions in reducing stress, promoting a sense of meaning, and improving quality of life among patients and families.

Several authors (Chochinov et al., 2005; Hall et al., 2013; Houmann et al., 2014; Kennedy & Essay, 2016) defend the need for interventions aimed at attenuating psychosocial and existential suffering and maintaining dignity at end of life.

According to Rosenfeld et al. (2017), the lack of meaning and purpose in life among PC cancer patients has been associated with the presence of high levels of distress. Likewise, Saracino et al. (2019) highlight the importance of psycho-existential interventions and therapies in PC, as they are mostly well established, empirically supported, and demonstrate improved outcomes among patients and families for relief of suffering, search for meaning, and promotion of dignity.

Therefore, it is vital that psychological interventions address spiritual and existential needs and seek to support the patients through life reflection and pursuit of meaning in life, enabling greater self-knowledge, maintenance of their identity and dignity (Saracino et al., 2019; Sousa, 2017; Warth et al., 2019).

In PC some psychotherapeutic interventions are especially effective in reducing spiritual and existential suffering and bolstering a sense of meaning, namely *Dignity Therapy* (TD), *Life Rev*iew (LR) and *Meaning-Centered Psychotherapy* (MCP) (OPP, 2019; Rosenfeld et al., 2017; Saracino et al., 2019; Sousa, 2017).

#### Dignity Therapy

*Dignity Therapy* is one brief, individualized and existential psychosocial intervention for use in patients approaching death, which has revealed a significant positive impact on attenuating psycho-emotional and existential suffering (Chochinov et al., 2005; Iani et al., 2020; Julião, 2014; Julião et al., 2013; Martinez et al., 2017; Warth et al., 2019). The main objective of this manualized intervention is to enhance the sense of meaning, purpose and dignity of terminally ill patients, allowing them to exchange affection and significant aspects of life, recall fond memories, solve unfinished businesses, and eventually create a legacy document to be bequeathed to family members (Chochinov et al., 2005, 2006, 2007; Chochinov & Kredentser, 2015; Espíndola et al., 2017; Julião et al., 2014, 2015; Vuksanovic et al., 2017).

The Model of Dignity in the Terminally Ill (MDTI), based on the experiences of patients diagnosed with cancer receiving palliative care, served as the theoretical basis for the *Dignity Therapy* (Chochinov, 2002). According to Chochinov (2012) and Julião (2014), this model provides a framework to guide health care professionals, patients and families in defining goals and therapeutic practices that enhance dignity at the end of life. The MDTI was based entirely on the reports of 50 cancer patients from palliative inpatient care programs (Hack et al., 2010). It consists of three main categories with general aspects that determine how patients experience dignity as death approaches, namely Illness Related Concerns, Dignity Conserving Repertoire and Social Dignity Inventory.

Illness-Related Concerns refer to how the illness affects the individual, such as physical and psychological responses, assessed by the level of independence (cognitive acuity and functional capacity) and by symptoms of physical distress (pain and discomfort) and psychological distress (medical uncertainty and death anxiety). Dignity Conserving Perspectives describes a set of spiritual and psychological factors that may influence a person’s sense of dignity. These resources are related to the patients' personal history, internal resources, personal actions, accumulated life experiences, as well as how they perceive the world and how these experiences shape their way of thinking and acting. They include eight subthemes: Continuity of Self, Role Preservation, Generativity/Legacy, Maintenance of Pride, Hopefulness, Autonomy/Control, Acceptance, Resilience/Fighting Spirit. Dignity Conserving Practices, which focus on Living “in the moment”, Maintaining normalcy and Seeking spiritual comfort. Finally, Social Dignity Inventory refers to the dynamics of patient-environment relationships, i.e., external resources. These resources - like internal resources - influence the sense of dignity through the social context and its positive and/or challenging events, and are assessed by privacy boundaries, social support, care tenor, burden to others, and aftermath concerns (Chochinov, 2012; Chochinov et al., 2002; Hack et al., 2010).

In Portugal, Julião et al. (2013) have conducted pioneering research on DT effectiveness. In their studies, they investigated the benefits of DT in alleviating symptoms of anxiety and depression and verified improvement in people with clinically significant psychological distress (Julião, 2014). DT proved to be effective in psychosocial suffering variables, namely demoralization, the desire to anticipate death, relief of suffering associated with loss of dignity, and improved quality of life.

According to Houmann et al. (2014) and Martinez et al. (2017), DT has shown clinically relevant results in several countries. However, the cultural context influences the implementation of psychosocial interventions. Although the therapy is effective, slight cultural adaptations are necessary to improve applicability (Houmann et al., 2014; Lindqvist et al., 2015; Miyashita et al., 2007).

Guo and Jacelon (2014) maintain that dying with dignity can be interpreted according to the individual’s history and a wider social and cultural context. Martinez et al. (2017) point out that the way a person faces the end of life can be an obstacle per se to the adaptation of DT. In addition to DT, other psycho-existential interventions are aimed at promoting dignity and psycho-existential well-being.

#### Life Review

*Life Review* is also a brief psychotherapeutic intervention designed to help people find meaning and purpose through a review of major themes and events in life. It consists of recalling, evaluating and integrating life experiences, leading the person, if possible, to resolve conflicts and create a sense of life completion (Huang et al., 2020; Vuksanovic et al., 2017).

A shorter version of LR – Short-Term Life Review – was proposed by Ando et al. (2008). It should last one week, include only two sessions and eventually prepare a formalized legacy document. Conversely, the extensive version of LR *–* Structured Life Review – lasts over four sessions and does not include a legacy letter (Ando et al., 2008).

Despite the dearth of therapeutic interventions, LR has proved to be beneficial in PC settings, as it has improved mood, self-esteem, life satisfaction, and perceived qualify of life in patients (Keall et al., 2015; Reischer & Beverley, 2019). It has further demonstrated a positive impact on the grieving processes of family members, as well as improvements in their spiritual well-being (Ando et al., 2010).

#### Meaning-Centered Psychotherapy

*Meaning-Centered Psychotherapy* is a therapeutic approach that aims to help people pursue meaning in life, spiritual well-being and quality of life. This approach has its theoretical basis in the work of Viktor Frankl, who suggested that human beings have the desire and ability to find meaning in life even in times of great suffering. Therefore, *Meaning-Centered Psychotherapy* sustains that PC patients can find meaning in life according to their choices and experiences, even in the face of the suffering associated with the disease (Rosenfeld et al., 2017; Thomas et al., 2014).

Both the *Meaning-Centered Group Psychotherapy* and the I*ndividual Meaning-Centered Psychotherapy*, when applied in groups or individually, respectively, have yielded significant psychosocial results, such as improved quality of life, reduced psychological distress, and enhanced spiritual well-being. Both versions of the *Meaning-Centered Psychotherapy* seek to provide a therapeutic space to assist patients in exploring personal issues and feelings related to the illness and suffering; to facilitate the understanding of the possible sources of meaning before and after the diagnosis of the disease; and ultimately, to help patients maintain a sense of meaning in life as the disease progresses (Saracino et al., 2019).

#### Resignification Process

*Dignity Therapy*, *Life Review* and *Meaning-Centered Psychotherapy* in PC share a common process of accessing narratives and subjective experiences of a person nearing death. The meaning individuals give to the events they experience throughout life are somehow related to the construction of their identity, with their understanding of themselves, and their behavior (Reischer & Beverley, 2019). It is, therefore, appropriate that individuals be accompanied in the task of narrating their stories, in order to build a more adaptive individual perspective on their existence, contributing to strengthening the sense of dignity and well-being (Reischer & Beverley, 2019).

### Forgiveness, Apology and Reconciliation

Forgiveness is one of the spiritual needs of human beings, as well as the need to love and be loved, their beliefs, a sense of purpose and life, and creativity (National Consensus Project for Quality Palliative Care, 2009).

It should be noted that reflection about life, relationships, evocation and evaluation of life events can trigger feelings of regret or need for self-forgiveness or being forgiven by others, by God or by a Supreme Being (Wittenberg et al., 2015). As human beings’ spiritual needs, forgiveness and reconciliation have been the subjects of innumerous studies about PC as they offer clinical relevance and critical points for reflection and intervention among patients and family members. Studies on spiritual care at the end of life (Leget, 2020; Renz et al., 2019) highlight forgiveness as an important topic to be addressed and managed to reduce personal suffering and guilt, strengthen relationships, and create peace of mind.

Therapies such as *Life Review*, *Reminiscence Therapy* and other techniques (e.g., active listening, verbalization of family conflicts, exploration of feelings of love, guilt or reconciliation, exploration of concerns and coping strategies) seem to facilitate forgiveness and reconciliation in PC (Silva et al., 2017).

According to Worthington (2005), forgiveness can be associated with better spiritual health, since the act of forgiving oneself and/or others and receiving forgiveness can provide a sense of liberation and inner peace. Forgiving can bring more satisfaction in life (Karremans et al., 2003), greater physical and emotional well-being (Ferrel et al., 2014), reduced feelings of guilt (Caldeira et al., 2017b; Vilalta et al., 2014), increased hope, optimism, and self-esteem (Maboea, 2003).

### Engendering Legacy

The creation of a legacy document as described in the studies by Vuksanovic et al. (2017) has demonstrated clinical relevance and significant positive impact on generativity, meaning and acceptance at the end of life. It is therefore suggested that the development of a document or final product that transcends death allows patients to have a new perception of their personal, family, social and professional heritage. It enables patients to guide future generations, to reappraise values, and to improve a sense of acceptance by integrating past and present events. McClement et al. (2007) showed that the creation of a legacy document can help maintain an adaptive grieving process. Furthermore, the authors refer to evidence of reduced levels of stress in family members, improved interaction between patients and others, and lower perceived physical symptoms.

Considering the paucity of investigations and resources of a psychological and existential nature in Portugal, the present pilot study intended to contribute to the development of a psycho-existential intervention adapted to the Portuguese cultural and social context, grounded in the *Dignity Therapy*, *Life Review* and *Meaning-Centered Psychotherapy*, integrating questions on the themes of forgiveness and reconciliation, and approaching PC as a holistic intervention that aims to reduce the suffering of terminally ill patients and their family members. Thus, a novel intervention protocol has been developed, which we named *Meaning of Life Therapy* (MLT).

## Method

The main goal of the present study was to develop the *Meaning of Life Therapy,* a novel psycho-existential intervention targeted at palliative care patients, and to determine its effectiveness and adequacy among Portuguese patients. Based on the analysis of the content of the generativity document, i.e., the *Life Letter*, produced by nine advanced cancer patients in this study, the following specific objectives can be outlined:

(i) To identify themes that emerge from the content of the patients' responses to the protocol developed;
(ii) To understand how the protocol questions facilitate a dialogue that guides the patient through a process of review, resignification and meaning-making of life events.
(iii) To investigate whether including questions about forgiveness, apology and reconciliation in the intervention protocol is appropriate and feasible and to understand its relevance for dignity, life review, resignification and meaning-making process among PC patients.

To achieve these specific objectives, the qualitative methodology appears to be the most adequate strategy. According to Lim et al. (2017), this methodological approach allows for the exploration of unknown phenomena and assessment of experiences in a holistic manner. It focuses on understanding the meanings given to the studied phenomena, based on the narratives and experiences of each individual, considering their contextual framework (Carrera-Fernández et al., 2014; Sutton & Austin, 2015).

### Participants

All patients attended the PC Outpatient Service of a hospital in northern Portugal, and were invited to participate if they had met the following inclusion criteria: (i) diagnosis of advanced cancer and with no expectation of a cure; (ii) 18 years and older; (iii) allopsychic and autopsychic orientation and cognitive acuity; (iv) insight into diagnosis and prognosis.

Twenty-five PC outpatient consultations at the hospital service were assessed. Thirteen patients met the inclusion criteria, who were invited and agreed to participate in the intervention. Nine patients completed the MLT protocol (Table 1).

**Table 1.** Sociodemographic and Clinical Characterization of Patients Undergoing the Intervention.

Code	Initial Diagnosis	Age	Gender	Main Caretaker	Marital Status	Schooling^a^	Time Since Diagnosis	ECOG PS^b^
“Aa”	Colon neoplasm	70	M	Son	Married	2nd cycle of basic education	1 Year and 4 months	2
“Bb”	Urothelial carcinoma of the bladder	69	M	Wife	Married	1st cycle of basic education	4 Years	2
“Cc”	Esophageal neoplasm	60	M	Daughter	Married	1st cycle of basic education	1 Year	2
“Dd”	Pancreas neoplasm	42	M	Wife	Married	Secondary education	2 Years and 1 Month	2
“Ee”	Gastric carcinoma	69	F	Daughter	Married	1st cycle of basic education	1 Year	2
“Ff”	Breast neoplasm	45	F	Husband	Married	2nd cycle of basic education	5 Years	2
“Gg”	Cervical carcinoma	41	F	Friend	Divorced	3rdCycle of basic education	1 Year and 2 Months	2
“Hh”	Lung neoplasm	47	F	Daughter	Married	3rd cycle of basic education	3 Months	2
“Ii”	Gastric carcinoma	72	F	Husband	Married	1st cycle of basic education	3 Years and 3 Months	2

^*^Eastern Cooperative Oncology Group Performance Status Scale 2: « Ambulatory and capable of all selfcare but unable to carry out any work activities; up and about more than 50% of waking hours» (Oken et al., 1982, p. 654).

^a^Basic Education in Portugal: first Cycle (Grades 1–4); second Cycle (Grades 5–6); third Cycle (Grades 7–9).

^b^Eastern Cooperative Oncology.

This is a convenience sample with five women and four men, with a mean age of 57.2 years and diagnosed with advanced cancer, followed up on at a PC outpatient or inpatient service. One participant withdrew the study and three participants failed to complete the intervention due to clinical deterioration and cognitive acuity impairment. All study participants live in northern Portugal, in both rural and urban areas.

### Materials

An intervention protocol was prepared with questions that intended to help patients find a sense of meaning and purpose in life and suffering; acquire greater self-knowledge; and preserve their identity and dignity through the recollection of autobiographical memories, review of themes and significant events, insights and guidance for future generations, and resolution of unfinished business, if the patient chooses to.

According to Callahan (2009), patients want to say goodbye to their family members; they need to feel forgiven and must have the opportunity to reconcile with loved ones. Therefore, two questions were included in this protocol that intended to explore the dying patients’ need for forgiveness, i.e., the need to offer and to ask for forgiveness or an apology. Other core values were included in the protocol in order to meet the overall psychological, existential and spiritual needs of PC patients. The concepts of forgiveness and apology were approached in two separate questions, therefore adapting the protocol to the needs and cultural components of Portuguese palliative care patients. Accordingly, the protocol included 14 questions (Table 2), which were subject to prior analysis by a specialist PC care team.

**Table 2.** Meaning of Life Therapy Intervention Protocol.

1. Which happy events would you like to remember?
2. What are the events in your life that you are grateful for?
3. What do you feel most proud of in life?
4. How would you like to be remembered by your family and friends?
5. What advice would you like to pass along to your family and friends?
6. Who would you like to ask for forgiveness or who would you like to be forgiven by?
7. Does anyone owe you an apology or do you feel that you owe someone an apology?
8. Is there anyone you would like to give a hug to?
9. Would you like to say goodbye to something or someone?
10. What have you learned about life?
11. What can make you happier now?
12. What can make your life worthwhile and give life a sense of meaning?
13. What do you think you still need to do?
14. How would you like this letter to be handled? Would you like to give it to someone? To whom and why?

At the end of the intervention, a generativity document, i.e., the *Life Letter*, was written by the psychologist, based on the responses given by the patients, who read the document, made any desired changes and finally approved it. Notably, this format facilitates life review and meaning-making processes in hopes of engendering a sense of generativity that is formalized in a document to be shared with loved ones. Thus, the *Life Letter* is able to promote communication between patients and family members at the end of life and during grief and bereavement.

### Procedures

#### Data Collection

A request for authorization was submitted to the Hospital da Senhora da Oliveira de Guimarães Ethics Committee (Nº11-2017) following a favorable opinion. All ethical and deontological considerations have been observed throughout the study, in compliance with the ethical principles involving research with human subjects defined by Declaration of Helsinki-Ethical Principles (World Medical, 2022).

The four-session protocol, as described by Warth, et al. (2019), was administered by one psychologist in cooperation with other psychologist of the team and the hospital PC professionals.

In the first session, objectives and procedures of the investigation were explained and biographical data, life history and preferences of the PC patient and families were collected. The content of the questions was clarified and the patient was given some time to reflect on the answers. Next, the patient was told that this protocol was aimed at creating a generativity document, i.e., the *Life Letter*.

After confirming the patient’s willingness to participate, the patient provided informed, written consent and completed a sociodemographic and clinical questionnaire. The second session usually coincided with the appointment scheduled at the hospital’s PC Service. The time between sessions was about one month. In the second session, the protocol questions were explored and the patient’s responses were audio recorded. Between the second and third sessions, the investigator fully transcribed the patient’s responses, and drafted the *Life Letter* to be presented in the third session.

In the third session, the draft of the *Life Letter* was given to the patient, who read, analyzed, and discussed its content with the investigator. They were able to suggest edits and make any desired modifications to eventually produce the final version of the letter. In the fourth and final session, the *Life Letter* was handed to the patient.

During seven months, nine patients participated in the four-session protocol, which was administered in a closed room to ensure privacy and data protection.

#### Data Analysis

A content analysis of the patient’s responses was carried out using a semi-inductive approach, since some themes had been previously defined based on the protocol domains, such as the exploratory questions about forgiveness and saying goodbye. However, other themes emerged from the data reflecting the participants' narratives about their life experiences and meanings in life. The first step was the open coding of text units, which were progressively grouped into sub-themes and themes (Saldaña, 2011). These were described and conceptualized using a constant comparative method (Glaser & Strauss, 1967), which involved two of the investigators. We tried to maintain the designation of the subthemes close to the language register of the participants.

## Results

Eight themes emerged from the content analysis of the *Life Letter*: (1) *Family*, (2) *Preservation of Identity*, (3) *Life Retrospective*, (4) *Clinical Situation*, (5) *Achievements*, (6) *Socio-Professional Valorization*, (7) *Forgiveness/Apology/Reconciliation*, and (8) *Saying* G*oodbye*. The themes, written in bold and italics in this section, included 30 sub-themes. Table 3 displays the themes according to the frequency of references in each theme. The themes (7) *Forgiveness/Apology/Reconciliation* and (8) *Saying Goodbye* were not included in this classification, as they are related to explicit questions of the intervention protocol.

**Table 3.** Description of General System of Categories.

Theme	Description	Subthemes	Participants’ Quotes
(1) Family (54 references)	It includes family-related aspects, roles played, emotional experience, markers in the patient’s family history	1. Recognition of the marital role	*“My husband is very good to me. My husband is very kind and helpful”*
		2. Recognition of the parental role	*“All my children are very good friends of mine”*
		3. Normative life cycle markers	*“The happiest event of my life was the birth of my children”*
		4. Fulfillment of family roles	*“My husband built our house. He would take our children with him and I would go to work to earn money to pay for food”*
		5. Closeness to family members and satisfaction in relationships	*“Being with my children and my husband is what can make me happy now”*
		6. Reinforcement of affections	*“For my daughters, to assure them that I love them |*referring to the recipient of the *Life Letter|. They know it already, but it would be another expression of love”*
(2) Preservation of identity (27 references)	It covers the individual characteristics and life perspective	7. Personal values	*“I’d like them to remember me as a fighter. An honorable person. A person who fights for what she wants”*
		8. Preservation of autonomy	*“If I have no more strength, life has no meaning…If I can carry on with my simple life, I am happy”*
		9. Continuity of self (through the maintenance of rituals and routines)	*“I have been to Santiago de Compostela. I have always felt very excited about these things. My husband told me that there was going to be an excursion and I said: “Let’s go!”*
		10. Acceptance	*“That´s life; there are good days and bad days. Life has its ups and downs, but that´s the way it is.”*
		11. Focus on the present	*“So, one day at a time. My own experience tells me this |* referring to saying Goodbye*|“*
		12. What keeps life meaningful (religious beliefs, personal identity, family ties)	*“Faith. If it wasn’t for it, I don’t think I would be here anymore”; “I think I have learned to be a better person since this happened to me! When I think about it, I feel accomplished”; “The love I have for my children and grandchildren”*
(3) Life retrospective (13 references)	It covers the way patients evaluate their life story	13. Valuing life as a whole	*“I have seen a lot in my life. I have made so many stupid things. I have enjoyed my youth. I have had many experiences. I have done so many crazy things, things you can´t even imagine. I think my life was worth living”*
		14. Valuing life before the illness	*“Because, to be honest, until I had the disease I was a happy person, I have never imagined that anything would happen to me. I feel grateful for all my life, which was happy until the onset of the disease. I don’t know if I was right or wrong to believe that the disease only happened to others. But only now did I start to think my life was good.”*
(4) Clinical situation (12 references)	It covers the patients’ current clinical situation, including illness-related needs and concerns	15. Treatment satisfaction	*“I am grateful to feel that I have good treatment for my problem.”*
		16. Achievements	*“Waking up after surgery… The doctor had told me that if everything went well, I would wake up at the Intensive Care. When I woke up and saw that I was at the Intensive Care, I didn’t know if it was day or night, but I felt a sense of relief.”*
		17. Uncertainties (related to disease evolution)	*“Everything went well, because if a minor problem had occurred, I wouldn’t have been here anymore, because I also know that the survival rate is very low”*
(5) Achievements (11 references)	It addresses the appreciation of material wealth acquired	18. Material wealth	*“I am grateful for what I have achieved. My husband and I worked hard to build a small house for us to live. It was on the 25th of April. He was building our house and he took our daughters with him and I went to work*
(6) Socio-professional valorization (5 references)	It addresses the appreciation of social roles	19. Friendship	*“Feeling that I have friends who make my life worth living, people who want the best for me. Feeling their support.”*
		20. Professional role	*“Having been a committed employee. I loved my work and I miss it. When I told my boss that I would be leaving, we both started crying. I was a good employee.”*
		21. Other roles in society	*“I feel grateful for everything I experienced with the scout group”*
(7) Forgiveness/Apology/Reconciliation	It covers the willingness or lack of willingness to resolve conflicts. The need to forgive and to apologize, or the desire to reconcile appear to be associated with the family context	22. No need to forgive (9 references)	*“I think no one”*
		23. No need to apologize (7 references)	*“I don’t think so… Not that I can remember! I’m not perfect, I may have failed at this or that, but I don´t think I owe anyone an apology.”*
		24. Apology or forgiveness from somebody (2 references)	*“I guess so. But if they think I’m waiting for it, I’m not.”*
		25. Desire for reconciliation (3 references)	*“I would like to hug my sister. I would like to have a conversation and reconcile with her.”*
		26. Emphasis on religious beliefs (3 references)	*“I would like to ask Jesus for forgiveness… And I would like to be forgiven by Him. It is by Him that I want to be forgiven”*
		27. Satisfaction with reconciliation (2 reference)	*“I have a sister-in-law who was angry at me and when I fell sick, she came to my house… I was very happy. One day we were at home and my brother asked me: “What would you like to eat?” I said I would like to eat a sardine pie and she brought me the pie. Even my children were happy with this gesture from my sister-in-law”*
(8) Saying goodbye [this theme combines the family dimension (desire to say goodbye to family members) and the clinical situation]	It covers the need and the lack of need to say goodbye to loved ones	28. Need to bid farewell (2 references)	*“To my children and my husband and my sister-in-law B. To my sister C”*
		29. No need to bid farewell (7 references)	*“I don’t think so… I think that when I’m approaching death, I think the less contact I have with someone, the better. I don’t think I’ll be able to face the situation without getting emotional.”*
		30. Uncertainty (as an obstacle to say goodbye) (3 references)	*“*|referring to the saying Goodbye*| This is a problem now. We don´t know how we will feel about it, when we are about to die”*

The theme *Family* (1) had the largest number of references – 54 references – in the participants’ narratives. This theme includes the patients’ insights regarding their roles played in the family. Patients highlighted the centrality of family relationships and the recounting the fondest memories within the family environment. According to the participants, these events contribute to the construction of their own identity, values, as well as a sense of purpose and dignity.

The theme *Preservation of Identity* (2) accounted for 27 references in the participants' narratives. It covers patients´ individual characteristics – values, attitudes and behaviors – the resources and strategies used to manage the present and past events and other experiences and challenges throughout their lifespan.

With regard to the theme *Life Retrospective* (3), which had 13 references, patients revealed how they perceive, attribute meaning and reappraise life events. This theme combines two perspectives: a whole-life perspective and a perspective on life before and after the illness. Participants recognized that some challenging events enabled them to reappraise values, adopt an attitude of greater acceptance, appreciate happy moments, and review some concerns.

The theme *Clinical Situation* (4) was mentioned 12 times in the participants’ narratives. This theme covers the needs and concerns reported by patients about their current clinical condition, namely disease progression, successful treatment outcomes, and patients’ experience from a physical and psychological point of view.

The theme *Achievements* (5), with 11 references, mainly refers to the appreciation of material wealth acquired throughout life. Participants not only recounted their efforts and accomplishments, but also highlighted a tangible legacy that would be left for their loved ones.

The theme *Socio-Professional Valorization* (6) was mentioned 5 times. It tackles the importance of personal, professional and community relationships for a sense of dignity and purpose. Similarly to the theme *Family* (1), participants recalled the contributions, relationships and roles played in their community and professional settings with gratitude and pride – although less often.

The theme *Forgiveness/Apology/Reconciliation* (7) refers to the manifestation of willingness or lack of willingness to resolve significant conflicts, to forgive or ask for forgiveness, and to give and receive an apology. The theme *Reconciliation* is related to the desire to reconcile and feelings of satisfaction after the reconciliation.

Forgiving or being forgiven, as well as giving and receiving an apology, may not necessarily imply the desire of all parties involved in the conflict; it may imply individual attitude alone. Conversely, a reconciliation effort requires the willingness and initiative of both conflicting parties. From the participants' perspective, forgiveness seemed to be largely related to “more serious” cultural conflicts or issues, so emphasis is given to religious beliefs or a Supreme Being. Apologies seemed to be associated with more common and culturally accepted mistakes. Participants reported that they did not feel the need to seek forgiveness (9 references), except God´s forgiveness (3 references). Two participants considered that some people owed them an apology and seven participants reported that they did not recognize the need to ask for an apology, although they might have disappointed someone – as part of human condition. There were three references to the willingness for reconciliation with someone or a Supreme Being. Two participants expressed satisfaction with the reconciliation.

Ultimately, the theme *Saying Goodbye* (8) is related to the need or a lack of need to bid a last farewell to loved ones. We noted that the theme *Saying Goodbye* was mostly associated with the last moments of life with their family members; it was not a continuous process related to how they lived or interacted on a day-to-day basis with the prognosis of a terminal illness. In fact, some patients mentioned that they would like to say goodbye to their closest relatives (2 references). However, most patients in this study maintained that they did not want to say goodbye to their loved ones (7 references), justifying that they would rather not think about that moment due to its emotional burden (4 references) or the uncertainty related to their medical condition, thus choosing to prevent family members from experiencing a scenario of significant physical or cognitive limitations (3 references).

The analysis of the *Life Letter* was intended to gain understanding of how the protocol questions facilitate life review, a meaning-making process and reappraisal of values. In the participants’ narratives, they disclosed and shared events, desires, wishes, concerns, needs, values and identity characteristics that they had prioritized throughout their lives before and after the diagnosis. We found that the protocol questions enabled patients to be involved in a dialogue that facilitated a new sense of meaning and purpose in life, based on the review of a set of events and fundamental values.

Thus, the opportunity to bequeath a letter to loved ones enabled patients to address affective bonds established throughout life and words of wisdom and guidance for future generations.

## Discussion

Psycho-existential interventions seek to respond to the patient’s psychological and existential needs and strengthen their sense of identity and continuity by recalling important events, substantiating values, and attributing a sense of meaning and purpose in life (Warth et al., 2019). These interventions have been the subject of several studies (Bauereiß et al., 2018; Huang et al., 2020; Rodin et al., 2020; Wang et al., 2017), which revealed positive effects in the psycho-spiritual and existential well-being of patients and their families.

The main goal of this pilot study was to develop a novel psychological intervention of psycho-existential nature (MLT), which was based on DT, MCP and LR. It seeks to meet the demographic, social, cultural, existential, religious specificities of the Portuguese population and the requirements of end-of-life care in Portugal. Accordingly, it proposes to discuss whether PC needs differ according to the cultural characteristics of the patients, which is often debated in the literature (Houmann et al., 2014; Keall et al., 2015).

The MLT intervention sought to combine the potentials and main goals of the therapies that served as a theoretical framework for the construction of the protocol questions, namely to promote dignity, to create a legacy document, and to carry out a life review.

The 14-question MLT protocol prompted patients to write a letter, in which they could address all their concerns and things they would like to recall, as well as include desires, wishes or life experiences that they considered charitable giving. This protocol cultivated a space for dialogue and reflection, in which patients disclosed significant events, memories, choices, decisions, personal characteristics, as well as relationships and bonds created, which were subsequently transcribed in a final document to be bequeathed to their family members. Adopting practices that seek to understand the psychological and existential needs of PC patients and reassessing them regularly is the core of individualized and patient-centered care in PC, which will enable the development of a comprehensive and adequate care plan to alleviate symptoms affecting quality-of-life (WHO, 2016).

The results of the present study revealed that the protocol questions facilitated the exploration of relevant psycho-existential themes among patients nearing death. By analyzing the content of the study participants' *Life Letter* we found that the aspects that the participants wanted to highlight usually reflected how they attributed a sense of meaning to life and suffering. This protocol was similar to the studies and interventions carried out with DT, LR and MCP by Vuksanovic et al. (2017). The themes addressed by the patients focused on the roles they played throughout their lives at family, professional and social levels, on the values and beliefs they preserved, and on their achievements and sense of completion. The patient’s relationship with their environments during their life course influences their sense of dignity, through the perception of the quality of established relationships and the emotional and social support received (Chochinov, 2002).

In the present study, it was possible to verify that family members were the primary source of emotional and support for the patients: in the past, through reciprocal affection and shared life, and though the key role played in meaning-making process and pursuit of dignity; in the present, through the emotional support and health care provided. Leading the patient through a process of reviewing and reappraising their lives using legacy document helps them develop a more authentic understanding not only of their identity but also of their life span and unity (Chochinov, 2012; Damon, 2021; Vuksanovic et al., 2017). According to Julião (2014) and Chochinov and Julião (2021), who have conducted several studies about DT, the exercise of creating a unique and subjective narrative of one’s own life reinforces the notion of generativity, which consists of the need and willingness to contribute to the world and younger generations.

Saracino et al. (2019) suggest that in PC, similarly to the Kübler-Ross recommendations, it is fundamental to use interventions based on existential principles, which involve the patient in an open discussion about their fears and concerns surrounding death and dying, and allow the patient and their families to maintain their dignity, find meaning, peace and purpose. In this sense, we consider that this protocol has proved to be a relevant brief psycho-existential approach.

With regard to the theme of *Forgiveness/Apology/Reconciliation*, in line with the studies conducted by Wittenberg et al. (2015) and Silva et al. (2017), we consider it important to reflect on these spiritual and existential needs in PC, in an attempt to facilitate giving and/or receiving forgiveness, offering and/or receiving an apology, to deepen feelings of love, and to resolve conflicts and/or unfinished businesses. Puchalski et al. (2009) found that spiritual issues (e.g., guilt) can lead to distress and cause psychological and physical suffering (e.g., depression, anxiety or acute pain). This theme is particularly relevant as it is associated with the patients’ individual characteristics, their religious beliefs, experiences and internal resources, which must be assessed in PC (Rego & Nunes, 2019). Health professionals should approach the patients’ spiritual and existential concerns using a biopsychosocial model (Chochinov et al., 2002), respecting and integrating the ethical limits of the relationship among psychologists, health professionals and patients. The questions of the MLT protocol deal with the themes of forgiveness, reconciliation and apology based on the studies by Post et al. (2000), who concluded that many patients want health professionals to address these issues. Thus, we believe that approaching and identifying this need, the patients may be provided spiritual care, if they choose to.

This protocol also included the opportunity to reflect on bidding farewell, which is not limited to the moment of death, but to a process that intended to encourage open communication on the subject, and to eventually create a generativity document that would facilitate this dialogue. According to Carvalho and Parsons (2012), saying goodbye is a spiritual need for patients nearing death, and it is up to health professionals to offer this possibility. In this sense, Melo et al. (2013) highlighted the importance of psychologists' interventions which should aim to understand finitude by promoting interventions that facilitate a dialogue between the patient, family members and PC team; to promote acceptance of the life they lived; to evaluate the support network; and to have the opportunity to say goodbye. In this study, it was verified that the questions related to the theme *Saying Goodbye* prompted an open discussion about issues less discussed between patients and family members. Thus, MLT can contribute to breaking the conspiracy of silence, a well-known challenge; to proposing solutions to subsequent problems, which are likely to cause suffering for the patient and family members; and later to supporting the grieving process of the family members.

Patients' responses to this theme depend on their personal history and internal resources. According to Bantim (2008), confronting the limitations of treatments and approaching the end of life activate suffering that requires internal organization. Therefore, we consider that, for some patients, these aspects are emotionally demanding, generating emotional discomfort and avoidance. To assist in this process, well-trained psychologists are needed to approach this issue.

### Implications for Practice

Considering that the Meaningful Life Therapy (MLT) protocol questions facilitate the exploration of relevant psycho-existential themes among participants, we believe that this new proposal presents itself as a reliable and promising therapeutic intervention for palliative care (PC) patients. The protocol questions enable a life review and meaning-making process by sharing significant events, desires, longings, concerns, needs, values, and identity, which patients have cherished throughout their lives, before and after diagnosis. Incorporating questions related to forgiveness, apology, and reconciliation provides patients with an opportunity to address conflicts and unresolved businesses, allowing them to find inner peace and create reconciliation with themselves, life, and others. Expressing wishes creates unique opportunities for the patients’ fulfillment, promoting a sense of well-being for the patients and a feeling of accomplishment among their relatives. All these elements, along with the opportunity to reflect on a final farewell, can open vital channels of communication among patients and family members, helping break the silence around end-of-life issues, which tend to be emotionally challenging and have harmful consequences. Compiling these items into a legacy document, i.e., the Life Letter, provides structure and organization to the therapeutic process of understanding one´s life. Finally, the Life Letter is an opportunity to bequeath a letter to loved ones, reflecting one’s life journey and purpose, as well as imparting wisdom and guidance for future generations. This can play a significant role in the process of saying goodbye, both for patients and family, and in supporting family members during the grieving process, as it offers an opportunity to remember the patient and “hear the his/her voice”.

For healthcare professionals, this therapy has the potential to introduce a novel approach, enabling them to better address the holistic needs—personal and familial needs—of PC patients. It implies the need for specialized training to foster a sense of confidence and competence in delivering PC care. Additionally, this concise intervention shows itself as a promising therapeutic tool for psychologists working with end-of-life patients. We understand that this intervention is able to make a significant contribution to the improvement of theory and practice in palliative care in Portugal. This is especially pertinent during its initial stages of implementation, addressing the unique needs and cultural dimensions of Portuguese palliative care patients and their families.

### Future Investigations

The current study has been tailored to address some of the fundamental psychological and spiritual needs and perspectives of PC patients. However, it has not encompassed other critical elements in this process, such as family members and healthcare professionals, to assess the intervention’s short- and long-term effects. Consequently, we recognize the importance of further research on MLT that considers these stakeholders, allowing for a comprehensive understanding of its potential effectiveness, particularly throughout the grieving process. In this context, a longitudinal analysis of the obtained results could provide input for the understanding of the effectiveness of the intervention over time. It may also be important to develop studies that allow a comparative analysis of MLT with other psychological interventions as well as its application to other populations in PC (e.g., neurodegenerative diseases). Additionally, it would be wise to offer precise definitions of concepts, such as forgiveness, apology, and farewell, within the context of palliative care (PC) patients and families, while adhering to the ethical standards of diverse PC teams. This clarification is crucial to promote a common understanding of these phenomena while considering cultural factors. In summary, the Meaningful Life Therapy represents an opportunity for advancing palliative care research in Portugal.

### Study Limitations

A drawback of this study was the long interval between MLT sessions. Notably, adapting and improving brief psycho-existential interventions in PC is paramount, given the rapid change in cancer patients receiving palliative treatment (Warth et al., 2019).

This study was further limited by challenges found during the implementation of the protocol related to the functioning of PC settings in Portugal: delayed referrals to PC services; the rapid degradation of the patients’ clinical situation; and consequently, the emotional and cognitive impairment and/or even death of the patient, making it difficult to proceed with or complete the intervention.

Finally, key issues in PC, such as denial and lack of insight about the palliative phase of the disease, responsible for the conspiracy of silence, also posed ethical challenges to the intervention.

### Conclusion

We believe that this study has provided a window into the development of brief PC therapies, as well as into research on psycho-existential interventions in PC in Portugal. It has proposed a novel intervention protocol – *Meaning of Life Therapy* – based on therapies with proven efficacy, which intended to meet the demographic, socio-cultural and PC service specificities in Portugal.

Implementing this intervention with PC patients allowed researchers to collect relevant data to adopt a continuous improvement model for PC services in Portugal through revision, manualization, adaptation, adequate education, and training of health professionals in PC.

Ultimately, this is a pilot study, and therefore further studies should be carried out to develop effective interventions that are evidence-based and culturally responsive to the idiosyncrasies of context, disease and actors (Saracino et al., 2019).
